# Comparative Effectiveness of Active and Reactive Mattresses in Pressure Injury Healing for Older People in Their Own Homes: A Pragmatic Equivalence Randomised-Controlled Study

**DOI:** 10.3390/nursrep15030111

**Published:** 2025-03-19

**Authors:** Katherine E. Rae, Judith Barker, Dominic Upton, Stephen Isbel

**Affiliations:** 1 Faculty of Health, University of Canberra, 11 Kirinari St, Bruce, Canberra, ACT 2617, Australia; 2 Canberra Health Services, Canberra Hospital, Yamba Dr, Garran, ACT 2605, Australia; 3 Faculty of Health, Charles Darwin University, Ellengowan Drive, Brinkin, NT 0909, Australia

**Keywords:** community, mattresses, pragmatic research, pressure injury, wound healing

## Abstract

**Background**: Pressure injuries are an ongoing problem commonly managed with the prescription of pressure mattresses. There is conflicting research about the comparable effectiveness of the two types of pressure mattresses, active and reactive. This, coupled with technological advances and an updated understanding of pressure aetiology, means decision-making when prescribing pressure mattresses is complicated. **Objective/Design**: A pragmatic approach was used to design an equivalence randomised-controlled trial investigating the comparative effectiveness of active and reactive pressure mattresses in a community setting from a wound healing perspective as well as from a user acceptability perspective. **Methods**: Participants with an existing pressure injury were provided with an active or reactive mattress for wound healing, with wound stages assessed using photography. Usual clinical care was provided based on the protocols of the health care service, including nursing and occupational therapy input. Participants were monitored for the healing of their existing pressure injuries, using the Revised Photographic Wound Assessment Tool. User acceptability feedback was provided through surveys, including impact on comfort, pain levels and bed mobility. An equivalence design was used for data analysis to determine if the surfaces were comparable. **Results**: Twelve participants completed the study, which found that people on active mattresses healed 11.71 days (95% CI −55.97–31.78 days) quicker than people on reactive mattresses; however, the small sample size meant that a definitive determination could not be made. Users found bed mobility more challenging, and pain levels decreased, regardless of mattress type. **Conclusions**: A pragmatic methodology is imperative for research in this field due to the complexity of pressure injury healing. Researchers exploring multi-faceted conditions should consider a pragmatic design to ensure transferability of results to the clinical setting. The results from this study were inconclusive when determining the equivalence of active and reactive mattresses due to the small sample size. When choosing a mattress, prescribers need to consider user preferences and mattress features to ensure user acceptability.

## 1. Introduction

The push for research that translates into practice is increasing, aiming to improve the uptake of evidence-based practice [1]. Pragmatic research methodologies, which consider real-world settings and multi-factorial elements of care, are well suited to this trend [1,2,3]. Despite pragmatic methodologies being introduced in 1967 [4], the focus in research has favoured explanatory designs, such as randomised controlled trials, to provide evidence-based health care [4,5]. Outcomes from explanatory studies, with strict eligibility criteria and testing conditions, can be challenging to replicate in clinical settings, raising questions about their real-world applicability [6].

This article explores the application of a pragmatic approach for research investigating the comparative effectiveness of active and reactive pressure mattresses on pressure injury healing in a community setting. The pragmatic approach was chosen due to the numerous factors influencing pressure injury development and healing [7].

Pressure injuries are a well-known global phenomenon that can have serious health and fiscal implications, costing the American public health system USD 26.8 billion annually, a figure which is avoidable mainly through pressure injury prevention techniques [8,9,10]. They can lead to wound healing complications such as multi-resistant infections and sepsis, as well as cause extended hospital stays, increased mortality, and psychological impacts such as anxiety and reduced quality of life [11,12]. Health care institutions prioritise pressure injury care to minimise to these costs [9,13,14].

The early understanding of pressure injury aetiology was that pressure injuries developed due to occlusion of blood vessels impacting tissue perfusion [15]. Pressure injuries would develop after prolonged periods of applied force, where tissue would begin to degenerate after some time from reduced blood flow. Research at the time was based on this assumption, with the design of pressure mattresses that emphasise temporary off-loading and encourage reactive hyperaemia as a means of tissue reperfusion. The understanding of pressure injury aetiology has changed in recent years, with a recognition that cell deformation causes pressure injuries rather than blood vessel occlusion [7,15,16]. This changed understanding implies that the impact of pressure on cells commences immediately after the application of force rather than after some time [15,16].

The provision of support surfaces such as active and reactive mattresses is well established for reducing the risk of pressure injuries, and healing developed pressure injuries [7]. Reactive mattresses adjust to load changes, while active mattresses redistribute pressure regardless of movement [7]. However, support surfaces are also expensive, so to ensure cost-effectiveness, prescribers must be aware of the characteristics of what they are prescribing and should be able to articulate the professional reasoning behind their recommendations.

In recent years, due to technological advances, reactive mattresses have been developed with an emphasis on reducing cell deformation at a surface level through immersion and envelopment, with suppliers reporting that these mattresses are equivalent to active support surfaces with regard to preventing and treating pressure injuries [17]. These reactive mattresses are often cheaper to purchase and easier to maintain due to their fewer mechanical parts. These factors are particularly relevant for the home environment, where maintenance of a complex mattress may be challenging or cost-prohibitive. However, research comparing the two different types of mattress design is often inconclusive or methodologically limited.

Research focusing on cost-effectiveness is challenging to compare due to the changing context in which pressure injury management occurs. Challenges include changes in costs as technology advances for equipment and wound care, and variability in health care systems regarding costs and policy. One example of this is evident when comparing two high-quality studies that both investigated the cost-effectiveness of mattress overlays compared with mattress replacements. Beeckman et al. [18] found that the reactive overlays investigated were a more cost-effective option than the active support surface used. In contrast, Nixon et al. [19] found that mattress replacements were more cost-effective than mattress overlays. These contrasting findings highlight the complexity and variability in determining the most cost-effective solutions for pressure injury management.

Research comparing the different types of mattresses has been primarily conducted in hospital and nursing home settings, where people are generally at very high risk of developing pressure injuries, either due to being acutely unwell or chronically unwell [17,20]. However, more frequently people are being cared for in their own homes and communities [21], meaning there is a wide variety of ‘healthiness’ in people who are at risk of pressure injury development—from people who are relatively well but with reduced mobility to those who are receiving palliative care in their own homes. Aloweni et al. [22] reported that although 70% of the patients identified with community-acquired pressure injuries used pressure-relieving devices, several factors complicated the use of support surfaces in the home. As a result, there is a great need for research conducted in domiciliary settings to explore and understand fully the unique challenges faced.

Clinicians in hospitals and residential care facilities have advantages in accessing and maintaining support surfaces. They possess product knowledge about the various surfaces used within the facility and have access to alternative options in the event of equipment failure [17]. In a hospital setting, when a malfunction occurs, a person can quickly receive a suitable replacement support surface due to systems that aim to reduce the incidence of pressure injuries [23]. However, the situation is more complex in a domiciliary setting. The availability of pressure mattresses and the physical capacity of the household members to move mattresses pose additional challenges. People using pressure-relieving equipment at home encounter unique difficulties related to assistive technology. These challenges may include troubleshooting issues due to less familiarity with pressure mattresses, power outages, and ongoing maintenance of the support surface [17].

These practical challenges significantly influence mattress choice as clinicians work with clients to avoid non-use of the mattress chosen [24]. For example, a clinician might recommend a non-powered mattress for someone living in an area with frequent power outages or recommend a mattress with a pump that is easy to operate and interpret when errors occur. Pragmatic research conducted in a community setting considers these complex contextual factors, enhancing the transferability of results from research settings to real-world situations. As a result, a randomised controlled trial was conducted to investigate the comparative effectiveness of active and reactive pressure mattresses in wound healing, exploring the equivalence of the mattress types.

### Research Questions

This study had two research questions:

1. Is a reactive mattress equivalent to an active mattress in healing an existing pressure injury when used in the domiciliary setting?
2. What is the acceptability of different mattresses to users in a domiciliary setting

Given this research was completed in the community, pragmatic challenges for this form of research were identified and reported, enabling recommendations for future community-based research in this area to be made.

## 2. Materials and Methods

**Figure 1:**
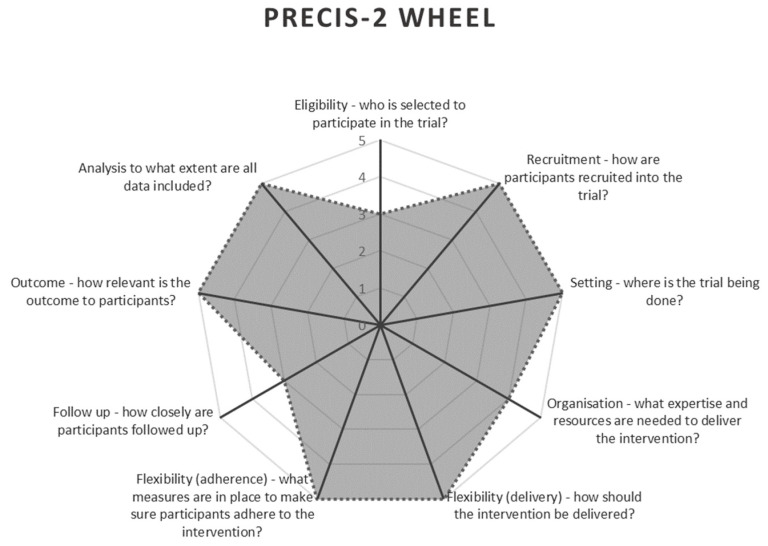
PRECIS-2 Wheel [25] highlighting the study’s pragmatic methodology aspects.

A randomised controlled trial with equal parallel groups was used for intervention allocation. The Pragmatic Explanatory Continuum Indicator Summary-2 (PRECIS-2) [25] was used to ensure the methodology aligned with the principles of pragmatic research. This tool scores the methodology along nine areas, with higher ratings indicating greater focus on pragmatics [25]. These nine areas are study eligibility, recruitment methods, study setting, organisation, flexibility of delivery of care, flexibility of adherence to protocol, follow-up, outcome measures, and analysis methods [25]. The PRECIS-2 wheel for this study is demonstrated in Figure 1.

An equivalence design was used rather than a superiority design. This design aims to show that the treatment group is equivalent to the control group rather than showing superiority [26,27] and aligns with the pragmatic method, “concerned with choosing the better mode of care rather than measuring a theoretical difference” [28] (p. 92). The pragmatic approach considered the complex nature of pressure injury treatment, influenced by numerous extraneous factors [4]. Site champions within individual regional community nursing teams were trained in the study protocols. These site champions facilitated participant recruitment and adherence to study protocols and acted as primary contacts for the research team [29].

### 2.1. Participants

Participants were recruited through the local public community health service, running a community care programme consisting of nursing and allied health services. Participants were identified as eligible for the study by community nurses or community occupational therapists, through referral or reporting of existing pressure injuries in the service’s risk management system. All participants who met the eligibility criteria were advised of the study by their treating health professional prior to being contacted by the research team to minimise selection bias. Participants were eligible for the study if they were aged 50 years or older, living in their own homes or supported independent living, sleeping in a bed (or were willing to return to sleeping in a bed for the duration of the study) and with an existing pressure injury of Stage 2, Unstageable or Suspected Deep Tissue injury without equipment already in place on the bed. These eligibility criteria reflect the most common clientele of the community health service [11,30]. They are similar to participants for research investigating pressure mattresses in residential care facilities and acute settings [18,19,31]. Clinical Practice Guidelines indicate that Unstageable pressure injuries can be re-classified once the wound bed is clearer [7]. At the research site, Unstageable pressure injuries were often re-classified as Stage 2 once the wound bed was clearer. If, upon re-classification, the wound were re-classified as Stage 3 or 4 then they ceased on the study. People with Stage 3 or 4 pressure injuries were excluded as these pressure injuries are less commonly seen in this setting and tend to have a much longer healing time, most likely skewing the results. People with Stage 1 pressure injuries tend to heal as quickly as three days [32], too quickly to show a difference in healing time between support surfaces due to the timeframes for the support surface reaching the individual.

### 2.2. Intervention

The research team randomly allocated participants to active or reactive mattress groups using a central random number generator to ensure concealed allocation. After baseline data collection, each participant received their allocated mattress set up on their preferred bed at home. Mattresses were categorised by pressure relief mechanism (immersion and envelopment versus temporary off-loading) rather than brand-specific features to replicate the variability of available mattresses in a community setting. Hybrid mattresses with both active and reactive modalities were excluded. Participants also received a high-profile air-floatation cushion for use when not in bed to ensure wound healing differences were primarily due to the mattress. These cushions provided the highest pressure injury risk reduction on variable chair surfaces [33]. For details regarding the provided support surfaces, see Table 1.

Apart from the provided support surfaces, participants received wound care and educational intervention based on the usual level of care provided per health service protocol developed using pressure injury clinical practice guidelines [7]. Community nurses completed wound care in ambulatory clinics or participants’ homes. Initially, wound care was provided daily, then reduced to second daily, and finally twice a week as the wound healed, following evidence-based protocols developed by the health care setting. Community occupational therapy was also provided for all participants, including a functional assessment for positioning based on the individual context and education on healing strategies and preventing future pressure injuries. This education, developed using the same guidelines [7], represented the standard level of care for the research site. Referrals to community allied health were made with participants’ consent following usual practices within the health care setting.

### 2.3. Outcomes

The primary outcome measure for comparative effectiveness was the time to complete healing [34]. Participants remained in the study until their pressure injury healed or they stopped using the allocated support surface (e.g., due to hospital admission). Healing was measured using the Revised Photographic Wound Assessment Tool, adapted from the Pressure Sore Status Tool [35]. The eight subscales of this tool include wound size and depth, wound edges, granulation and necrotic tissue, and peri-ulcer skin status. This tool has high validity and reliability compared with in-person assessment [36] and reflected the pragmatics of the community practice setting. Photographs taken at each wound change allowed for the blinding of assessors, as it was impossible to blind participants or clinicians due to visible differences in mattresses. The photographs were assessed and scored collaboratively by a minimum of two tissue viability advisors, trained in the use of the tool by the research team. The data from photographs at baseline and at the point of healing were used in the analysis.

Patient-reported outcome measures included comfort and ease of mobility, assessed using a 7-point Likert scale (with higher scores indicating greater comfort/ease of movement) and pain levels assessed using a 10-point pain scale with descriptors for each level (higher scores indicating more pain). These scales were part of two surveys that also gathered subjective information regarding ease of use, personal opinions on the mattress’s strengths and weaknesses, and any troubleshooting required. The research team developed the surveys in consultation with expert clinicians in the practice setting. They were completed at baseline and at a two-week follow-up to allow participants to adjust to the new mattress. Additional information was collected to ensure group similarity, including general demographics (age, gender and co-morbidities), the participant’s Waterlow Scale score [37] for baseline pressure injury risk level, movement patterns, time spent in bed, and adherence to pressure injury prevention strategies such as regular skin checks, skin care, and repositioning.

### 2.4. Sample

Sample size calculations could not be completed due to the lack of available data regarding standard deviations (SD) in the previous literature exploring pressure injury healing. Reviewed articles either did not use similar outcome measures or did not report the SD due to non-normal distributions. The sample size was determined using the central limit theorem [38], aiming for a total sample of n = 80 to achieve a minimum final sample of n = 60 (n = 30 per group) after allowing for withdrawals.

Due to visible differences in the mattresses, participants and treating clinicians could not be blinded to the intervention. To reduce bias, photographs of the pressure injuries were assessed by tissue viability advisors who had no clinical involvement with the participants and thus were blind to the allocated support surface [39].

### 2.5. Statistical Analysis

Statistical analysis was used Cumming’s guiding principles of New statistics [40], which explores data on a pragmatic level, looking at estimation methods beyond null hypothesis testing. Kruschke and Liddell’s New Bayesian statistics [41] were used for the technical approach (region of practical equivalence). Bayesian estimation aims to derive the probable values of unknown model parameters by integrating information from the data (likelihood) and existing knowledge (prior) [42]. This method was used due to the variable nature of pressure injury healing. Unlike frequentist methods, Bayesian approaches do not rely on large-sample approximations and instead use the prior to enhance computational stability through regularisation [41]. Consequently, Bayesian methods are well suited for small sample sizes. Data analysis was conducted using RStudio (version 2022.12.0+353.pro20), including the RStan and brms packages for the survival analysis.

For this study, the region of practical equivalence was set at ±7 days, determined through expert consensus for a minimal clinical difference. Subject matter experts from Europe, America and Australasia with occupational therapy, nursing or research backgrounds were consulted [43]. Twenty-five experts were approached via electronic correspondence, with a 52% response rate. Weibull survival analysis was used to compare the healing times between the two groups, and generalised linear models were used to compare scores relating to comfort, pain levels and ease of movement in bed.

The aim was to complete both Intention to Treat (ITT) analysis and Per-Protocol (PP) analysis, as an ITT analysis tends to favour the study hypothesis in equivalence studies [44]. Since all eligible participants received their allocated intervention and completed the study to an endpoint, ITT and PP analyses were identical, and all available outcome data were included. Missing data were due to the pragmatic nature of the data collection.

### 2.6. Trial Registration and Ethics

This study and protocol was registered with Australian New Zealand Clinical Trial Register (Registration No ACTRN12618000319279, registered on 5th March 2018). Ethical approval was received from ACT Health Services Human Research Ethics Committee (ETH.10.17.233) and University of Canberra Human Research Ethics Committee (Approval No: 20180161), with all participants providing written informed consent following discussion with the research team. This study was conducted and complied with Good Clinical Practice (GCP) Guidelines. No adverse events were recorded in this dataset. One adverse event was recorded in the pilot dataset that, upon review, was attributed to factors outside to the study.

## 3. Results

**Figure 2:**
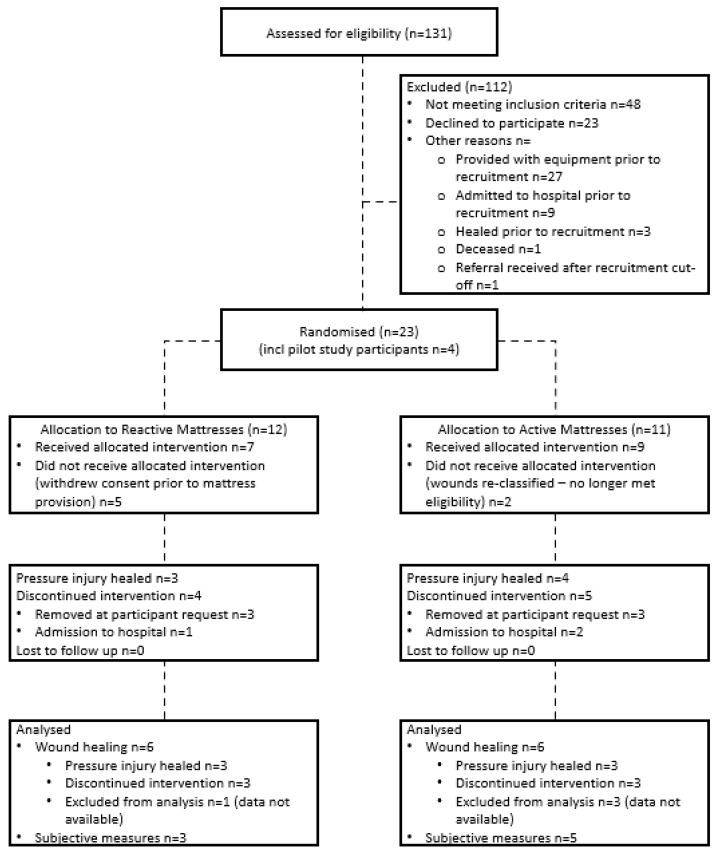
Study Flow.

A total of nineteen participants were recruited during this study. Their data were aggregated with participants from an earlier pilot study [45] as the eligibility criteria for the current study extended those of the pilot study, with no changes to the intervention protocol. This resulted in a total of 23 participants across both datasets. Seven participants withdrew prior to receiving their allocated mattresses: two due to their wounds being re-classified and no longer meeting eligibility criteria and five withdrawing consent prior to mattress provision. This resulted in sixteen participants in total, with nine allocated to active mattresses and seven allocated to reactive mattresses (Figure 2). Due to missing photographs, final wound care data were available for twelve participants (active:reactive—6:6). Complete survey data were available for 50% of participants (n = 8, active:reactive—5:3). All participants received and completed the intended treatment. Therefore, Intention to Treat and Per Protocol analyses were identical, with the study protocol and data analysis accommodating participants whose pressure injuries did not heal before their completion of the study.

Recruitment for the study took place from March 2019 to April 2022, with the final participant follow-up completed in May 2022. The study ended due to timeframe issues related to ongoing recruitment challenges following COVID-19, including increased pressure on the health system and participant concern as a vulnerable population. As a result, the target sample size was not achieved.

Characteristics for the two participant groups can be found in Table 2. Participants in the reactive group were at slightly higher risk of pressure injury development, with higher mobility scores indicating more restricted mobility. Pressure injuries were primarily located at the sacrum or heel, with other locations consisting of the ischial tuberosity (1× active, 2× reactive) and the hip (1× active). All participants with Unstageable pressure injuries or Suspected deep tissue injuries were monitored and re-classified as Stage 2 pressure injuries once a clearer assessment could be made.

### 3.1. Comparative Mattress Effectiveness for Pressure Injury Healing

**Figure 3:**
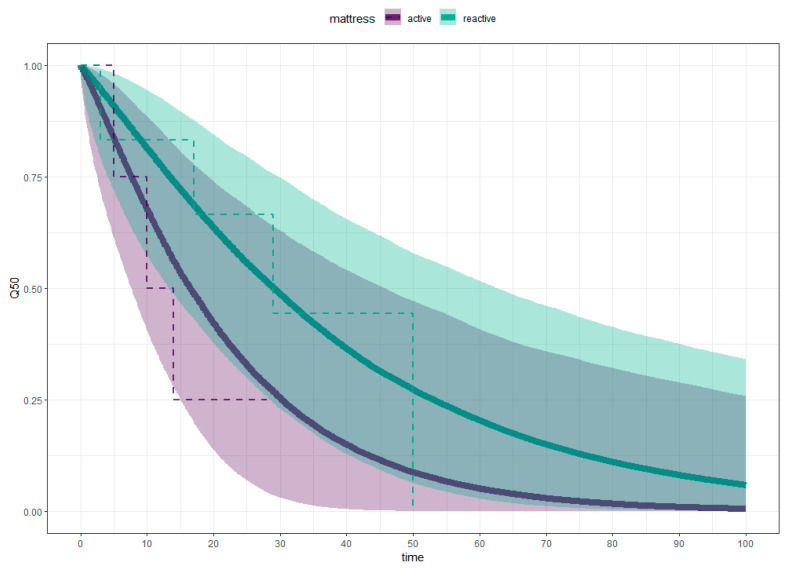
Weibull Survival Analysis (solid line), Kaplan–Meier Estimate (dotted line).

**Figure 4:**
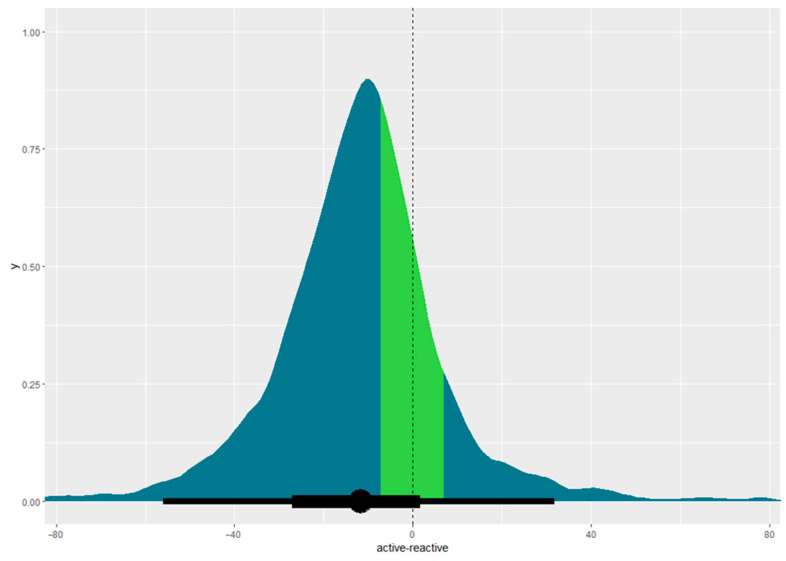
Equivalence density curve with equivalence margin demonstrated as 95% CI. Coloured stripe indicates the region of practical equivalence of ±7 days, with the dotted line indicating the midpoint of the region of practical equivalence.

Participants on active mattresses healed quicker than those on reactive mattresses. Using the 50% survival point as a reference, people on active mattresses healed in 16.54 days (95% CI, 6.40–59.09 days) compared with 29.20 days (95% CI, 11.12–75.55 days) for those on reactive mattresses, as can be seen in Figure 3. The difference in healing times between active and reactive mattresses is −11.71 days (95% CI, −55.97–31.78 days), outside the equivalence margin of ±7 days, as shown in Figure 4. Figure 4 illustrates the equivalence density curve, with the stripe indicating the equivalence interval of ±7 days. The curve is positioned to the left, indicating the shorter healing times for the active mattress group for this dataset. Due to the small sample size, these results may not represent the true population, as evidenced by the overlapping credible intervals in Figure 3 and the wide 95% CI straddling the region of practical equivalence in Figure 4.

### 3.2. Comparative Mattress Effectiveness for User Acceptability

Regarding subjective factors, survey data for ease of movement, changes in pain levels and comfort levels were collected. Participants also provided feedback about what they liked and disliked about the support surface.

**Figure 5:**
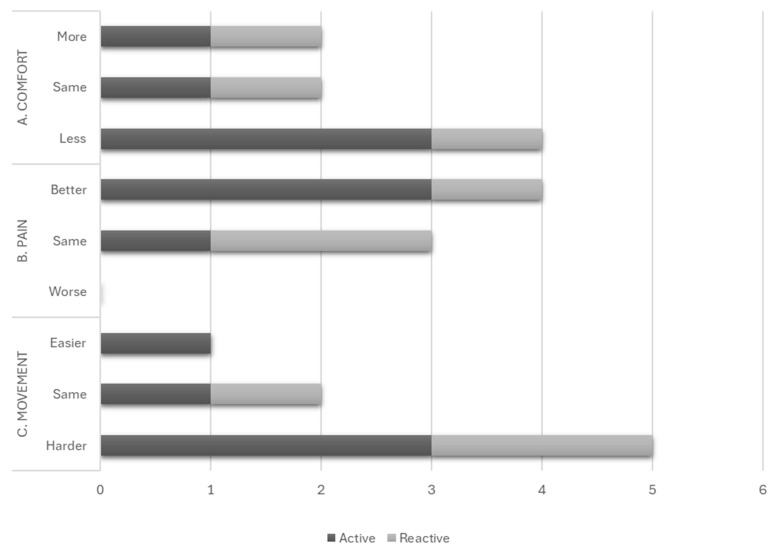
Subjective factors of user acceptability: (**A**) Comfort, (**B**) Pain and (**C**) Movement.

More participants found the active mattresses less comfortable than their usual mattresses compared to the reactive mattress group (Figure 5A). More participants on active mattresses reported improved pain levels than those on reactive mattresses (Figure 5B). Most participants found their allocated mattress challenging to move on or the same as their usual mattress, with only one person finding it easier to move on their allocated active mattress than their usual mattress (Figure 5C).

Beyond the subjective ratings reported in Figure 5, participants described their experiences with the provided mattresses. Notable challenges included the noise of the pump (active mattresses only), the size of the mattress affecting shared bed space, and the perceived temperature of the mattress. Some relevant quotes appear below:

*“The sound of the pump woke me frequently … When the changes in the pressure inside the mattress happened, there were noises that woke me.”*

(Participant X4)

*“It takes up too much room on our double bed, making hardly any room for my husband”*

(Participant 16)

*“I found it cold to sleep on, so put a sheepskin and blanket on top.”*

(Participant 2P)

## 4. Discussion

This study utilised a pragmatic design to assess whether reactive mattresses were equivalent to active mattresses in healing pressure injuries when used in the domiciliary setting and to gauge user acceptability of these mattresses in such settings. Research outcomes for pressure mattress studies often use clinical efficacy, aligning well with pragmatic outcomes. However, existing studies tend towards explanatory methodologies that may not reflect standard practice [46]. The pragmatic methodology aligns with the multifaceted aspects of pressure injury healing and the range of available support surfaces. The use of this methodology is consistent with the literature describing research conducted in similar, complex settings beyond pharmaceutical research [3].

### 4.1. Equivalence of Mattress Types

Past research on mattress efficacy for pressure injury management has been inconclusive due to the variety of mattresses and the complexity of care [47,48]. Most randomised-controlled trials use superiority tests due to the way research questions are framed (explanatory intervention is better than control intervention). However, in clinical practice, the focus is more on ensuring the explanatory intervention is not inferior or at least equivalent to the control intervention. However, when an explanatory intervention has a non-significant result, the results are often misinterpreted as being inferior [49]. Equivalence tests allow for an interpretation that aids practice–that the interventions are non-inferior or equivalent. Bayesian statistics, coupled with visual data presentation, aids accurate data description and reduces misinterpretation risks.

The results of this pragmatic study were inconclusive regarding whether a reactive mattress is equivalent to an active one for healing pressure injuries. Figure 4 shows a large discrepancy between the equivalence interval and the results, with the 95% CI substantially straddling the region of practical equivalence, indicating high levels of uncertainty. The substantially overlapping credible intervals in Figure 3 suggest the sample was too small for definitive conclusions. This does not imply the superiority of active mattresses but highlights the need for more data on healing times for a valid comparison [26].

### 4.2. User Acceptability

Bayesian statistics use established knowledge, known as the prior, to inform the data analysis [50]. Participants evaluated known acceptability factors influenced by pressure mattresses—ease of movement, comfort and impact on pain levels [7,11]. Difficulty moving in bed aligns with the expected immersion and envelopment of support surfaces, even active surfaces [7]. Pain levels remained similar or reduced across mattress types, as anticipated, as support surfaces are designed to alleviate pain resulting from pressure injuries [11]. Given the highly individual preferences and the small sample size, further research is necessary to better understand the different facets of user acceptability [51]. Prescribers should involve patients when prescribing mattresses to minimise equipment abandonment and improve patient outcomes. Likewise, researchers should integrate consumer feedback to ensure interventions meet the intended needs of the participant.

### 4.3. Pragmatic Considerations

Pressure injury management is a complex intervention due to the many variables that can influence the development and healing of a pressure injury. Research designs investigating one factor of complex interventions such as pressure injury management must consider all the intrinsic and extrinsic factors that will influence an individual’s risk level. When exploring the transferability of research into clinical practice, pragmatics must be considered to ensure that all these separate but interacting components are scrutinised [4]. However, it is not realistic to control all the variables [52]. A pragmatic research design is a good way of considering these factors without controlling them [4,53]. A pragmatic design also considers the limitations of the practice setting, where resources are often more restricted than in a funded research setting [53]. However, by maintaining the pragmatic focus, challenges often arise in the methodology [4,53]. The challenges experienced in this study relate to the practice context, recruitment, data analysis and data collection [6,29,54].

#### 4.3.1. Practice Context

This study was conducted in a domiciliary setting utilising existing health services for interventions administered in the participant’s home. Working in a domiciliary environment poses challenges due to the variability within each home [55]. Unlike clinical settings with the required resources, a domiciliary setting lacks consistent lighting and assistive technology, making good care more complex [55]. The study’s setting impacted the photography used for tracking the wound progress, with issues such as poor lighting and unstable camera positioning. However, by adhering to a standard health service protocol, there are high levels of transferability of the results for other domiciliary-based settings.

#### 4.3.2. Recruitment

Recruitment challenges in clinical trials are well-known. For pragmatic trials, challenges often stem from the health professionals’ experience, available resources, and patients’ complex co-morbidities [6,29]. This study encountered difficulties related to both factors.

Recruitment was impacted by COVID-19, causing direct delays due to lockdown periods, decreased participant confidence, and increased burden on the health service. The pandemic exacerbated the existing challenge of adhering to trial protocols in a clinical setting [29]. Although site champions were used to enhance recruitment and facilitate communication, their impact was diminished by staff turnover and unprecedented health care pressures.

The Risk Management system was also used to identify candidates, with the research team seeking consent for contact through the treating team [6]. Delays in system entries led to many potential participants receiving equipment before recruitment. This tension between providing optimal care and protocol adherence is a recurring issue in pragmatic trials [29], as seen by the distribution of pressure-relieving equipment.

In explanatory research, people with co-morbidities are often excluded [6], particularly in studies involving older populations, where multiple factors impact outcomes. People with pressure injuries often have additional health concerns that increase the risk of pressure injury development and impact wound healing [56]. Pragmatic research aims for representative samples, including people with co-morbidities [6]. However, this posed a challenge in our study as participants were often admitted to health care facilities before or during the study. By ensuring that a possible exit point was an individual no longer using the mattress, we ensured that the data collected would still be useable, employing statistical methods that accommodated censored data.

#### 4.3.3. Data Collection

When researchers collect data, the resources available, such as additional time, are manageable. However, in pragmatic research, where clinicians often collect data, resources such as extra time for data collection are limited, so clinicians prioritise outcomes for individual patients over the research outcomes [46]. This conflicting priority can present challenges regarding recruitment and protocol adherence, further impacting the validity of the research if not managed [6,29].

Performance bias is a common challenge for pragmatic studies focusing on complex interventions because the therapeutic relationship is an integral component of the intervention [52]. Performance bias was mitigated by adhering to the health services’ evidence-based pressure injury protocol. However, the interaction between clinicians (nurses and occupational therapists) and the study participant may vary in other interventions, such as the frequency of the wound dressing or follow-up education [52].

Wound assessment photographs, traditionally used for monitoring healing, are increasingly being utilised to blind research assessors [57]. Considerations of use include lighting, camera specifications, and manual handling due to injury site and participants’ frailty [58]. In this pragmatic study, photographs taken with smartphones at the participant’s home, resulted in lower quality images despite standardised photography techniques. High-quality, well-lit images that clearly show peri-ulcer skin are ideal [59]. Health organisations should establish photography protocols for optimal image capture, including skin preparation, lighting, and camera stabilisation.

#### 4.3.4. Data Analysis

Pragmatic studies have high external validity due to the nature of the design. However, this can increase threats to internal validity [2]. Although effort was put in to maintain high levels of internal validity, the small sample size obtained means that the study’s external validity is impacted. Using a parametric model also allows for more stability with the smaller sample size, aiming to minimise the impact on external validity. Data analysis models were chosen to maximise the stability of the statistical model. It is acknowledged that Kaplan–Meier estimators provide descriptors of the existing data [60]. However, they describe the data as it is available and cannot provide a prediction for censored data, such as leaving the study prior to wound healing [61]. Weibull exponential models are an appropriate replacement for Kaplan–Meier estimators [60]. A Weibull model provides a means to adjust variables and make comparative inferences about the healing times between the two groups when there is censored data [61]. However, it must be stressed that the results from this study are not definitive concerning the effective comparison. The considerable overlap of credible intervals in Figure 4 visually represents the high level of uncertainty.

### 4.4. Limitations

The primary limitation of this study was the small sample size. Despite initial projections suggesting a higher incidence of pressure injuries, subsequent health system changes and challenges in identifying candidates promptly resulted in a smaller cohort. This reflects a broader issue in non-pharmaceutical randomised-controlled trials, where achieving adequate sample size is challenging [5,62], further bringing into question the emphasis on the explanatory design. Future studies could utilise a pragmatic, observational design using existing data from clinical records, such as wound images and mattress prescriptions, to investigate comparative effectiveness between the mattress types.

### 4.5. Recommendations for Future Research

Research must bridge the gap to clinical practice, which poses numerous challenges for research project design [4]. Pragmatic research aids the exploration of complex interventions by encompassing the diverse factors that may influence an outcome, making findings more applicable to real-world practice and thus facilitating evidence-based practice [4,63]. Adoption of pragmatic research design is expected as the call for evidence regarding complex and nuanced interventions increases across the health sector. However, research design must be comprehensive to be truly pragmatic. Tools like PRECIS-2 [25], will help to consider all elements of pragmatic design. The following should be considered when designing a pragmatic study:

- *Aligning study protocols with clinical practice* is crucial for maintaining adherence, especially when competing priorities exist [29,63]. “Trials that are embedded in routine care and require limited additional activities pose fewer barriers for health care professionals and patients to participate” [6], p. 177. For example, if infrared technology for early identification of pressure injuries is not standard at the study site, its inclusion could hinder protocol adherence and limit transferability to settings without such technology.
- *Consider data collection by treating clinician or research team.* While data collection by research teams improves protocol adherence, it is resource-intensive, particularly when operating over multiple research sites [6]. Conversely, clinician-led data collection is more time-efficient; however, it may have lower adherence due to competing care priorities and less familiarity with the protocol [6]. Balancing these factors is essential for pragmatic studies in complex health care systems [6]. The degree of variation from the clinical practice of the settings can guide decision on who should collect data.

## 5. Conclusions

In an increasingly complex world, pragmatic research ensures that results remain clinically relevant and replicable. The study of support surface efficacy for pressure injuries is inherently complicated by numerous factors that impact wound healing and daily life variables. Pragmatic research is well placed for such studies, accounting for these complexities in its conclusions. Despite uncertainties regarding the clinical equivalence of active versus reactive mattresses, further research using pragmatic design promises more transferable findings. In the meantime, clinicians should consider the individual factors, such as the individual’s functional mobility and environmental elements, when prescribing support surfaces to meet specific individual needs.

## Figures and Tables

**Table 1:** Support Surfaces.

Active Mattresses	Reactive Mattresses	Cushions
Novis BetterLiving APMBL-101 (MO) Novis Premier Digital 5 (MO) CuroCell A4 CX16 (MR) Novis Premium 9 (MR)	ROHO (MO) Softform Premier (MR) Novis Cairmax (MR)	High profile ROHO single valve High profile Star single valve

MR = mattress replacement, MO = mattress overlay.

**Table 2:** Participant Demographics.

Characteristics	Active Group (n = 9)	Reactive Group (n = 7)
Age (mean, (standard deviation)) Gender (male:female)	82.89 years (9.83) 7:2	79.00 years (4.83) 4:3
Medical Co-morbidities (*n*, (%)) Cardiovascular Neurological Pulmonary Diabetes Incontinence Malnutrition Other	6 (66.67%) 1 (11.11%) 3 (33.3%) 3 (33.33%) 5 (55.57%) 2 (22.22%) 4 (44.44%)	6 (85.71%) 3 (42.86%) 2 (28.57%) 1 (14.29%) 1 (14.29%) 2 (28.57%) 4 (57.14%)
Pressure injury location (*n*) Sacrum/coccyx Heel Other	6 1 2	3 2 2
Pressure injury Stage (*n*) Stage 2 Unstageable Suspected deep tissue injury	7 1 1	6 0 1
Waterlow Scores (mean (standard deviation)) Total score at baseline Mobility Body	19.33 (7.40) 1.78 (1.30) 1.78 (1.30)	19.71 (4.54) 3.00 (1.53) 1.71 (1.60)
Pressure Injury Prevention Time spent in bed (mean (standard deviation)) Skin checks Moisturising skin Movement/Repositioning	7.33 h (3.39) o o +	9.20 h (1.92) o + +

+ strong adherence, o moderate adherence, - weak adherence.

## Data Availability

The data that support the findings of this study are available on request from the corresponding author. The data are not publicly available due to ethical restrictions.

## Public Involvement Statement

Public involvement occurred during the development of the pre- and post-treatment surveys, to ensure the questions were understandable to a lay person and covered areas that were relevant for the patient.

## Guidelines and Standards Statement

This manuscript was drafted against the CONSORT Guidelines for pragmatic research.
